# *SOCS3* Methylation Predicts a Poor Prognosis in HBV Infection-Related Hepatocellular Carcinoma

**DOI:** 10.3390/ijms160922662

**Published:** 2015-09-18

**Authors:** Xin Zhang, Qingshan You, Xiaolei Zhang, Xiangmei Chen

**Affiliations:** 1 Department of Radiotherapy, Harbin Medical University Cancer Hospital, Harbin 150081, China; E-Mail: zhangxin1262008@126.com; 2 Department of Microbiology and Infectious Disease Center, School of Basic Medical Sciences, Peking University Health Science Center, Beijing 100191, China; E-Mails: xiaoleizhang8802@163.com (X.Z.); xm_chen6176@163.com (X.C.)

**Keywords:** hepatitis B virus, hepatitis C virus hepatocellular carcinoma, methylation, prognosis, *SOCS3*, translational start site

## Abstract

Suppressor of cytokine signaling 3 (*SOCS3*) plays crucial roles in JAK/STAT signaling pathway inhibition in hepatocellular carcinoma (HCC). However, the methylation status of *SOCS3* in HBV infection-related HCC and the relationship between *SOCS3* methylation and the clinical outcome remain unknown. Here, we reported that in HCC tumor tissues, two regions of the CpG island (CGI) in the *SOCS3* promoter were subjected to methylation analysis and only the region close to the translational start site of *SOCS3* was hypermethylated. In HCC tumor tissues, *SOCS3* showed an increased methylation frequency and intensity compared with that in the adjacent non-tumor tissues. Moreover, *SOCS3* expression was significantly down-regulated in HCC cell lines and tumor tissues, and this was inversely correlated with methylation. Kaplan–Meier curve analysis revealed that in patients with an hepatitis B virus (HBV) infection background, *SOCS3* hypermethylation was significantly correlated with a poor clinical outcome of HCC patients. Our findings indicated that *SOCS3* hypermethylation has already happened in non-tumor tissues and increased in both frequency and intensity in tumor tissues. This suggests that the methylation of *SOCS3* could predict a poor prognosis in HBV infection-related HCC patients.

## 1. Introduction

Hepatocellular carcinoma (HCC) is a common malignancy worldwide with rapid progress and high short-term mortality. The initiation of HCC is intimately associated with chronically diseased liver tissue, induced by etiological factors such as hepatitis HBV or hepatitis C virus (HCV) infection, toxin exposure, excessive alcohol consumption, and/or other environmental or genetic factors [1]. Over 80% of HCC cases in China are attributed to chronic HBV infection [2,3]. Although surgical excision is still the most effective treatment so far, a large proportion of HCC patients show symptoms of intrahepatic metastases or post-surgical recurrence [1]. Therefore, the identification of more sensitive and specific biomarkers associated with HCC prognosis is essential to improve HCC management in China [4].

In the liver, the sequential progression to carcinoma is a multistep process with an accumulation of genetic and epigenetic alterations leading to the activation of oncogenes and the inactivation of tumor suppressor genes (TSG) [5,6]. DNA methylation, which is the first epigenetic regulation, has been suggested as one of the most important molecular mechanisms in hepatocarcinogenesis [5,7]. Mounting studies have revealed that HCC tumors exhibit specific DNA methylation signatures that are associated with major risk factors and tumor progression [8]. However, the methylation status of TSGs in HCC with different virus infection, especially HBV infection, is not fully understood.

The suppressor of cytokine signaling (*SOCS*) gene family has been found to play essential roles in suppressing tumor progression by inhibiting JAK/STAT, NFκB signaling, and promoting p53 signaling [9,10,11,12]. Among the *SOCS* family, *SOCS3* has been reported to be hypermethylated in various kinds of cancers, including head and neck cancer, lung cancer, prostate cancer, Barrett esophagus carcinoma, and ulcerative colitis-related colorectal cancer [13,14,15,16,17,18,19]. In HCC, Niwa *et al.* revealed that *SOCS3* was hypermethylated in 33.3% (6/18) of HCC tissues [20]. However, in our previous report, we did not observe *SOCS3* hypermethylation in HBV-related HCC tissues [21]. These conflicting results may be due to the complicated etiological mechanism of HCC and the different detected loci in the *SOCS3* promoter region. Therefore, it is imperative to explore the detailed methylation status of *SOCS3* in HCC with different virus infection backgrounds and the relationship between its methylation and clinicopathology.

In this study, the methylation status of two loci of the CpG island (CGI) in the *SOCS3* promoter was quantitatively investigated, with particular attention to the change in methylation intensities in primary HCC tissues with different virus infection backgrounds. Also, the clinicopathological significance of *SOCS3* methylation status was statistically analyzed.

## 2. Results and Discussion

### 2.1. Results

#### 2.1.1. Suppressor of Cytokine Signaling 3 (*SOCS3*) CpG Island (CGI) Is Hypermethylated in the Region Close to the Translational Start Site

**Figure 1:**
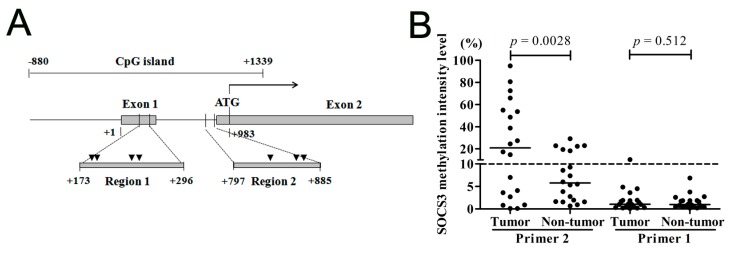

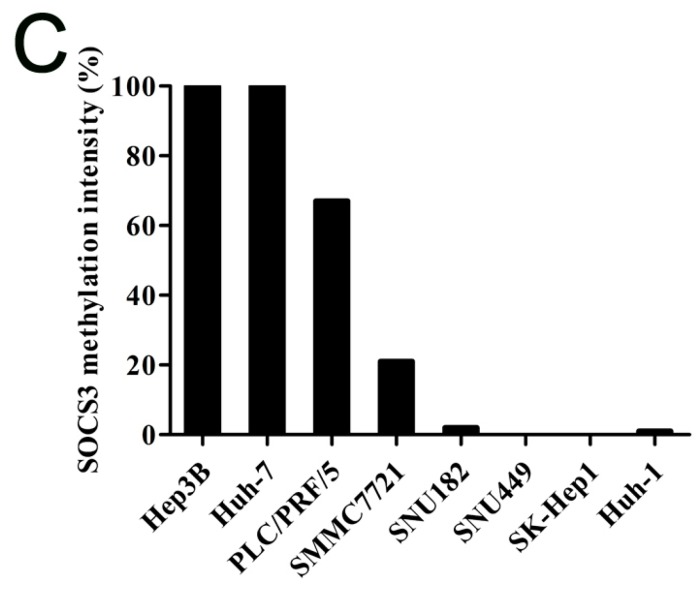
The methylation statuses of two loci of the *SOCS3* promoter in HCC tissues and cell lines. (**A**) Schematic representation of the two detected CGI loci of the *SOCS3* gene. The transcriptional start site for *SOCS3* gene is defined as +1. Shaded boxes are exons of *SOCS3*. “▼” represents the restriction enzyme cutting site; (**B**) The methylation statuses in the located regions of Primer 1 and Primer 2 in 20 pairs of tumors tissues and the adjacent non-tumor tissues; and (**C**) The methylation status of *SOCS3* at region 2 in eight HCC cell lines.

Using the online University of California Santa Cruz (UCSC) database, we searched for CGI at the *SOCS3* promoter region and one CGI surrounding the *SOCS3* gene promoter was predicted. This CGI region starts from −880 nt and ends at +1339 nt, covering the translation initiation site of the *SOCS3* gene. In our previous study, we detected the methylation status in the CGI region from +173 to +296 nt (region 1) in HCC tissues and did not find any methylation in this region. However, Niwaet *et al.* observed obvious methylation in the CGI region in HCC tissues, which was close to the translational start site (Figure 1A). To investigate whether the above conflicting results were due to the different detected regions in the *SOCS3* promoter, we designed another pair of primers to amplify the CGI region from +797 to +885 nt (region 2), which was close to the translational start site and near the locus reported in the Niwaet *et al.* report [20].

Firstly, we detected the methylation statuses of both region 1 and region 2 in 20 pairs of the HCC tumor and non-tumor tissues. With the cut-off value setting at 10%, any tissue that had a hypermethylation intensity ≥10% would be judged as the hypermethylation status, and the higher value implicated a higher CGI hypermethylation status in intensity [15]. As shown in Figure 1B, at target region 2, 12 of the 20 tumor tissues and six of the non-tumor tissues showed hypermethylation. The methylation intensity in HCC tumor tissues was also found to be significantly higher than that in the adjacent non-tumor tissues (*p* = 0.0028). However, the methylation status at region 1 showed no hypermethylation in both tumor and non-tumor tissues. Next, we detected the methylation statuses of both regions in eight HCC cell lines. We found that region 2 of the *SOCS3* CGI was hypermethylated in cell lines Huh-7, Hep3B, PLC/PRF/5, and SMMC7721, while it was unmethylated in SNU449, SNU182, SK-Hep1, and Huh-1 cells (Figure 1C). Similar to that in HCC tissues, no methylation was detected in region 1 in all these cell lines (data not shown). These results indicated that it was the locus close to the translational start site that showed increased methylation, rather than that near the transcription start site.

#### 2.1.2. *SOCS3* Methylation Is Associated with Altered *mRNA* Expression in Hepatocellular Carcinoma (HCC) Cell Lines

**Figure 2:**
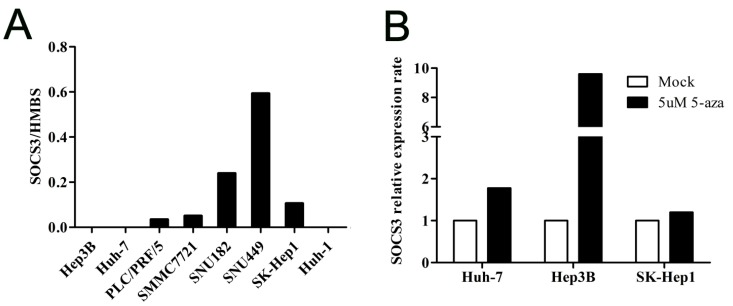
The *mRNA* expression of *SOCS3* in HCC cell lines. (**A**) The *mRNA* expression levels of *SOCS3* in eight cell lines; and (**B**) the normalized *mRNA* expression levels of *SOCS3* in cell lines Huh-7, Hep3B, and SK-Hep1 after treatment with 5-Aza-2ʹ-deoxycytidine for three days.

To further investigate whether the hypermethylation of region 2 in the *SOCS3* CGI could suppress *SOCS3* gene expression, the expression levels of *SOCS3* in these eight HCC cell lines were quantitatively measured by real-time qPCR. As shown in Figure 2A, the *SOCS3* *mRNA* expression level was relatively lower in cell lines with high *SOCS3* CGI methylation such as Huh-7, Hep3B, PLC/PRF/5, and SMMC7721. In contrast, in cell lines with *SOCS3* CGI non-methylation, such as SNU449, SNU182, and SK-Hep1 cells, the *mRNA* expression of *SOCS3* was relatively higher. Next, we processed Huh-7, Hep3B, and SK-Hep1 cells with the treatment of *DNA* demethylation reagent 5-aza-2ʹ-deoxycytidine. We found that after 5-aza treatment, the mRNA levels of *SOCS3* significantly increased in Huh-7 and Hep3B cells with hypermethylation, but not in the SK-Hep1 cells with non-methylation (Figure 2B). These data suggest that the methylation in CGI region 2 is associated with the down-regulation of *SOCS3* expression in HCC cell lines. In addition, we noticed that the *mRNA* expression level of *SOCS3* was also very low in Huh-1 cells with non-methylation, suggesting that besides methylation modulation, there might be other regulation mechanisms of *SOCS3* expression, such as acetylation [22].

#### 2.1.3. *SOCS3* CGI Is Hypermethylated in Primary HCC Tumor Tissues

**Figure 3:**
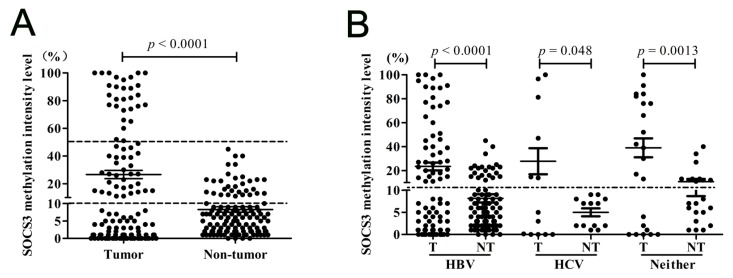
The methylation statuses of *SOCS3* at region 2 in HCC tumor and adjacent non-tumor tissues. (**A**) The methylation status in 127 enlarged paired tumor tissues and non-tumor tissues; and (**B**) the methylation status in tumor tissues and adjacent non-tumor tissues with different virus infection backgrounds.

Next, we detected the methylation status of region 2 in *SOCS3* CGI in an enlarged HCC sample. In the paired 127 HCC specimens, 61 (48.03%) of the tumor tissues and 32 (25.21%) of the adjacent non-tumor tissues showed methylation (*p* = 0.0002), which meant that in non-tumor tissues, the *SOCS3* methylation had already happened. Meanwhile, the hypermethylation intensity in tumor tissues was significantly higher than that in the adjacent non-tumor tissues (26.63% *vs.* 8.31%, *p* < 0.0001, Figure 3A). In addition, in tumor tissues, there were 26.77% (34/127) specimens in which he *SOCS3* methylation intensity was over 40%, while there were only 2.36% (3/127) in non-tumor tissues.

To test whether the *SOCS3* CGI methylation in HCC tissues was associated with different virus infections, we analyzed the methylation frequency and intensity among 92 pairs of HCC tissues with HBV infection, 13 pairs with HCV infection, and 22 pairs with neither HBV nor HCV infection. In these three groups of HCC, the methylation frequencies in tumor tissues were 44.57% (41/92), 46.15% (6/13), and 63.63% (14/22), respectively; and in the adjacent non-tumor tissues, 25% (23/92), 0 (0/13), and 45.45% (10/22). Additionally, the methylation intensity of tumor tissues in each group was significantly higher than that in their corresponding non-tumor tissues (*p* < 0.0001, *p* = 0.048, and *p* = 0.0013). However, no marked difference was found among the tumor tissues with different virus infection backgrounds (Figure 3B). Taken together, these results suggested that the frequency of *SOCS3* CGI hypermethylation in HCC tissues was not related to the different virus infection backgrounds of HCC.

#### 2.1.4. *SOCS3* Is down-Regulated in Primary HCC Tumor Tissues

**Figure 4:**
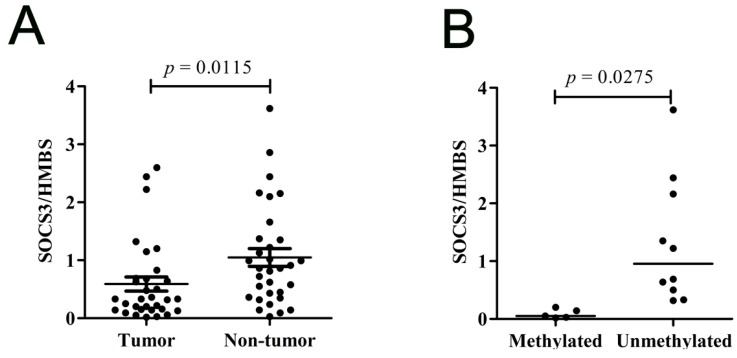
*SOCS3* *mRNA* expression levels in tumor and non-tumor tissues. (**A**) *SOCS3* *mRNA* expression level in 32 pairs of HCC tumor and non-tumor tissues; and (**B**) *SOCS3* expression level in hypermethylated HCC tissues (intensity ≥ 40%) and unmethylated tissues (intensity < 3%).

We also detected *SOCS3* *mRNA* expression levels in 32 pairs of HBV-related HCC tumor tissues and non-tumor tissues (Figure 4A). As expected, the level of *SOCS3* *mRNA* in tumor tissues was statistically lower than that in non-tumor tissues (*p* = 0.0115). Among these 32 tumor tissues, the *SOCS3* methylation intensity in five tumor tissues was extremely high (≥40%) and 10 specimens unmethylated (<3%). We found that the expression level of *SOCS3* in tumor tissues with extremely high *SOCS3* methylation was significantly lower than that in the tumor tissues without methylation (Figure 4B). Comparing the *SOCS3* expression levels of these two groups, they was inversely correlated with the CGI hypermethylation status. These data further demonstrated that the down-regulation of *SOCS3* expression in HCC tissues was associated with *SOCS3* methylation.

#### 2.1.5. Methylation of *SOCS3* Is Associated with the Poor Prognosis of HCC with Hepatitis B Virus (HBV) Infection

We then statistically analyzed the association between *SOCS3* hypermethylation and patient clinicopathological features in HCC cases. As shown in Table 1, *SOCS3* methylation was not correlated with the tumor size, tumor-node-metastasis (TNM) stage, cirrhosis background, portal vein tumor thrombosis, and tumor encapsulation of the HCC patients. It was worth noting that in the older group of HCC patients (≥55 years), the methylation frequency of *SOCS3* in HCC tissues was higher than that in the younger group (<55 years). However, the difference of *SOCS3* methylation between younger and older HCC groups was not statistically significant.

**Figure 5:**
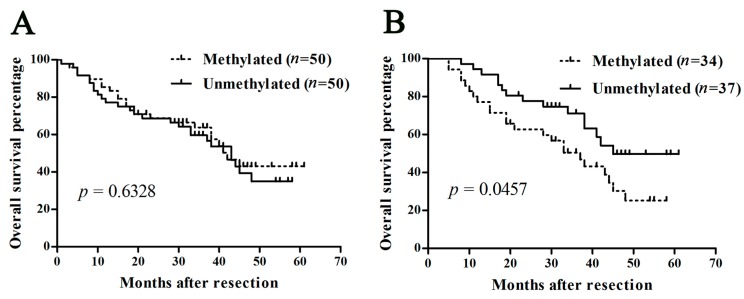
Kaplan–Meier survival plots for *SOCS3* methylation in HCC patients. Overall survival after surgery according to *SOCS3* methylation status in (**A**) general HCC patients and (**B**) HBV infection-related HCC patients.

We then evaluated the prognostic value of the *SOCS3* methylation status in HCC patients. The Kaplan–Meier curve analysis indicated that, for all the patients, there was no difference in clinical outcome between the methylated and unmethylated patients (*p* = 0.633, Figure 5A). However, in patients with an HBV infection background, *SOCS3* hypermethylation was significantly correlated with the poor prognosis of HCC patients (*p* = 0.0457, Figure 5B), which meant that HCC patients with *SOCS3* hypermethylation exhibited shortened overall survival compared with those with *SOCS3* unmethylation, while no correlation was found in the rest of the patients with HCV or no virus infection background (data not shown).

**Table 1:** *SOCS3* CGI hypermethylation and clinicopathological correlations in HCC.

Feature	Hypermethylation (*n* = 61)	Unmethylation (*n* = 66)	*p*-Value
Gender			
Male	46 (52.87%)	41 (47.13%)	0.107
Female	15 (37.5%)	25 (62.5%)	
Age			
≥55	42 (54.5%)	35 (45.5%)	0.068
<55	19 (38%)	31 (62%)	
Cirrhosis			
Yes	47 (47.47%)	52 (52.53%)	0.813
No	14 (50%)	14 (50%)	
TNM stage			
I–II	22 (48.89%)	23 (51.11%)	0.886
III–IV	39 (47.56%)	43 (52.44%)	
Portal vein tumor thrombosis			
Present	9 (40.91%)	13 (59.09%)	0.267
Absent	52 (50.98%)	50 (49.02%)	
Tumor size			
≥5 cm	46 (47.92%)	50 (52.08%)	0.964
<5 cm	15 (48.39%)	16 (51.61%)	
Tumor encapsulation			
Complete	52 (50%)	52 (50%)	0.800
Incomplete	6 (37.5%)	10 (62.5%)	

### 2.2. Discussion

The aberration of *SOCS3* in human malignancy has been noted in recent years, but the detailed methylation status of *SOCS3* in HCC with different virus infection backgrounds has not been well understood. Here, we confirmed that *SOCS3* was hypermethylated in HCC tissues and *SOCS3* hypermethylation was significantly correlated with the poor prognosis of HCC patients with HBV infection.

Using Methylation-sensitive restriction enzyme-based quantitative PCR (MSRE-qPCR) assay, we found that it was the CGI region close to the translational start site of the *SOCS3* gene that was frequently hypermethylated in HCC tumor tissues and cell lines. *SOCS3* hypermethylation in HCC tissues was not related to the etiological mechanism of HCC. However, *SOCS3* CGI methylation was associated with poor prognosis in HCC with an HBV infection background, while it was not associated with poor prognosis in HCC with HCV or no virus infection background, which indicated that *SOCS3* might play a more powerful role in HBV infection-related HCC; the detection of the methylation of the *SOCS3* gene was of value for the prognosis of HCC with HBV infection. Additionally, we also found that *SOCS3* CGI hypermethylation occurred partially in non-tumor tissues, although its intensity and frequency were far lower than those in tumor tissues. Since most of these HCC patients had a cirrhosis background, it was reasonable to suppose a potential oncogenic role for *SOCS3* hypermethylation during hepatocarcinogenesis from as early as the precancerous cirrhotic stage. Furthermore, no methylation was found in non-tumor tissues in HCC patients with HCV infection, suggesting that different hepatitis viruses might have different effects on the methylation status of *SOCS3*.

Aberrant methylation of gene promoter regions is one of the mechanisms for TSG inactivation in cancers. The decreased expression of *SOCS3* has been reported to be correlated with poor patient survival in HCC tissues [23]. We also observed the frequent down-regulation of *SOCS3* *mRNA* levels in HCC cell lines and tumor tissues, which was inversely associated with the *SOCS3* methylation status. In addition, treatment by the demethylation agent could restore *SOCS3* expression in the methylated cell lines. These findings confirmed that methylation modulation was an important inhibiting factor for *SOCS3* expression. However, in Huh-1 cell lines and some HCC tissues with low expression of *SOCS3*, no methylation was detected, which indicated that other mechanisms could also lead to the down-regulation of *SOCS3*, including mutation, acetylation, and *microRNA* regulation, *etc.*

It has been reported that through inhibiting the JAK/STAT signaling pathway, the *SOCS* gene family plays an important role in suppressing tumor initiation and development [14]. As an essential member of this gene family, *SOCS3* may function as a tumor suppressor gene. In previous reports, *SOCS3* down-regulation was associated with vascular invasion and overall survival [24,25]. The silence of *SOCS3* expression facilitated tumor formation and growth in lung and liver [26], while the loss of *SOCS3* could promote aggressiveness in hepatocellular carcinoma [27]. However, the underlying mechanism of *SOCS3* in the pathogenesis of HCC remains elusive. Further study should be conducted to determine the precise intracellular signaling and explore potential mechanisms of *SOCS3* in the initiation and development of HCC.

In conclusion, our findings suggest that it is the CGI region close to the translational start site that is methylated in HCC, which might contribute to the aberrant expression of *SOCS3*. The hypermethylation of *SOCS3* has already happened in non-tumor tissues and increased in tumors in both frequency and intensity. *SOCS3* CGI methylation was associated with poor prognosis in HCC with a HBV infection background. However, it still needs a larger cohort to confirm this result.

## 3. Experimental Section

### 3.1. Tissue Samples

Primary HCC and the adjacent non-tumor tissues were obtained from 127 patients who underwent routine curative surgical treatment at Henan Cancer Hospital in Zhengzhou, Henan Province, China, from 2008 to 2014. Among the 127 patients, 92 patients were infected with HBV, 13 patients with HCV, and 22 patients with no virus infection background. All patients were diagnosed by ultrasonography and computed tomography, and confirmed by liver biopsy. No patients had received radiotherapy or chemotherapy prior to surgery. The 127 patients were histologically diagnosed with HCC. The clinicopathological characteristics were shown in Table 2. The follow-up data were described in detail previously [28]. The study protocol was approved by the institute ethics committee, and informed consent was acquired from all patients and donors before the study commenced.

**Table 2:** Clinicopathological parameters of 127 patients with HCC.

Clinicopathological Parameter Variables	Cases *n* (%) *n* = 127
Age	
Median (Range)	57 (11–80)
Gender	
Male	87 (68.50)
Female	40 (31.50)
Liver cirrhosis	
Yes	99 (77.95)
No	28 (22.05)
TNM stage	
I–II	45 (35.43)
III–IV	82 (64.57)
Portal vein tumor thrombosis	
Present	22 (17.32)
Absent	102 (80.32)
N/A	3 (2.36)
Tumor size	
≥5 cm	96 (75.59)
<5 cm	31 (24.41)
Tumor encapsulation	
Complete	104 (81.89)
Incomplete	16 (12.60)
N/A	7 (5.51)

All patients were from the local ethnic Chinese Han population. TNM: Tumor, Lymph Node; Metastasis; and N/A: Not available.

### 3.2. Cell Culture and 5-Aza-2ʹ-Deoxycytidine Treatment

Human HCC cell lines SMMC7721, Huh-1, Huh-7, Hep3B, SNU449, SK-Hep1, SNU182, and PLC/PRF/5 were kept in our laboratory and maintained in either Dulbecco’s Modified Eagle Medium (DMEM) or Roswell Park Memorial Institute (RPMI) 1640 supplemented with 10% fetal bovine serum (GIBCO, Carlsbad, CA, USA). For 5-aza-2′-deoxycytidine treatment, cells were seeded in six-well plates at a concentration of 2–2.5 × 10^5^ cells per well and treated with 2 μM of 5-aza-2ʹ-deoxycytidine for three days.

### 3.3. DNA Extraction and Methylation Assay

Genomic *DNA* was isolated using DNeasy Blood & Tissue Kit (Qiagen, Germantown, MD, USA) and was quantified using NanoDrop 2000 spectrophotometer (Thermo Scientific, Waltham, MA, USA). MSRE-qPCR was performed to detect the methylation status of *SOCS3* as previously described [29]. The methylation primers of *SOCS3* were as follows: Primer 1, forward: AGCTCGAGGGACGCGCGCGCGAA, reverse: CTGAGCCCCCTCGGGTC-CCCAAG; Primer 2, forward: GGTGCCCCAGTCGCTCGGCGAAG, reverse: GGAAGGCG-CGAGCGCGTTGAGTGC. The cut-off value was set at 10%, as previously report [21].

### 3.4. RNA Extraction and Real-Time RT-qPCR

Total *RNA* was prepared using total RNA isolation (TRIZOL) reagents (Invitrogen, Carlsbad, CA, USA) in accordance with the manufacturer’s instructions. The expression level of *SOCS3* was measured through the real-time RT-qPCR assay using the Roche lightcycler 480 (Roche, Mannheim, Germany) according to the manufacturer’s instructions. The house-keeping gene *HMBS* was included in the assay as control reference to normalize the expression levels, and primers of *HMBS* have been described previously [30]. The *SOCS3* qPCR primers were as follows: forward: GGAGACTTCGATTCGGGACC; reverse: GGTACTCGCTCTTGGAGCTG. Each experiment was conducted in duplicate or triplicate, and the comparative *C*_t_ method (2−∆*C*_t_) was adopted to determine the relative expression levels of *SOCS3*.

### 3.5. Statistical Analyses

The differences between groups were analyzed by two-tailed paired or unpaired *t*-test. The χ2 test or Fisher’s exact test were adopted to determine the association between the *SOCS3* methylation status and the clinicopathological features. The Kaplan–Meier analysis with log-rank test was used to analyze the primary liver cancer (PLC) prognosis and survival time. All of the statistical analyses were performed by GRAPHPAD PRISM software (GraphPad Software, Inc., San Diego, CA, USA). In all cases, a *p*-value of less than 0.05 was considered significant.

## 4. Conclusions

Our findings suggested that *SOCS3* hypermethylation has already happened in non-tumor tissues, while its frequency and intensity were increased in tumors. The methylation of *SOCS3* predicted a poor prognosis in HBV infection-related HCC patients.
